# Dynamics of Hierarchical Urban Green Space Patches and Implications for Management Policy

**DOI:** 10.3390/s17061304

**Published:** 2017-06-06

**Authors:** Zhoulu Yu, Yaohui Wang, Jinsong Deng, Zhangquan Shen, Ke Wang, Jinxia Zhu, Muye Gan

**Affiliations:** 1 Institute of Applied Remote Sensing and Information Technology, College of Environmental and Resource Sciences, Zhejiang University, Hangzhou 310058, China; yuzl@zju.edu.cn (Z.Y.); 21614113@zju.edu.cn (Y.W.); jsong_deng@zju.edu.cn (J.D.); zhqshen@zju.edu.cn (Z.S.); kwang@zju.edu.cn (K.W.); 2 State Key Laboratory of Hydroscience and Engineering, Department of Hydraulic Engineering, Tsinghua University, Beijing 100084, China; 3 Institute of Land and Urban-Rural Development, Zhejiang University of Finance & Economics, 18 Xueyuan Road, Hangzhou 310019, China; jxzhu@zufe.edu.cn

**Keywords:** urban green space, landscape metric, Landsat, greening policy

## Abstract

Accurately quantifying the variation of urban green space is the prerequisite for fully understanding its ecosystem services. However, knowledge about the spatiotemporal dynamics of urban green space is still insufficient due to multiple challenges that remain in mapping green spaces within heterogeneous urban environments. This paper uses the city of Hangzhou to demonstrate an analysis methodology that integrates sub-pixel mapping technology and landscape analysis to fully investigate the spatiotemporal pattern and variation of hierarchical urban green space patches. Firstly, multiple endmember spectral mixture analysis was applied to time series Landsat data to derive green space coverage at the sub-pixel level. Landscape metric analysis was then employed to characterize the variation pattern of urban green space patches. Results indicate that Hangzhou has experienced a significant loss of urban greenness, producing a more fragmented and isolated vegetation landscape. Additionally, a remarkable amelioration of urban greenness occurred in the city core from 2002 to 2013, characterized by the significant increase of small-sized green space patches. The green space network has been formed as a consequence of new urban greening strategies in Hangzhou. These strategies have greatly fragmented the built-up areas and enriched the diversity of the urban landscape. Gradient analysis further revealed a distinct pattern of urban green space landscape variation in the process of urbanization. By integrating both sub-pixel mapping technology and landscape analysis, our approach revealed the subtle variation of urban green space patches which are otherwise easy to overlook. Findings from this study will help us to refine our understanding of the evolution of heterogeneous urban environments.

## 1. Introduction

Urbanization is dominated by population growth, industrialization, and land resources, and has been associated with a series of environmental issues, such as heat islands [1], urban flooding [2], and habitat loss [3]. Urban green space (UGS) is represented by all vegetation cover in and around cities, including urban forest, grassland, parks, green roofs, gardens, urban farms, and street trees, and is widely believed to provide critical ecosystem services and play a vital role in mitigating the negative impacts brought about by urbanization [4]. These include air pollution reduction [5], storm water runoff interception [6], energy use reduction [7], noise reduction [8], and habitat preservation [9]. Moreover, the presence of UGS is believed to provide numerous psychological benefits, such as relieving stress and anxiety [10] and promoting social cohesion [11]. Studies have also emphasized the importance of aesthetic views and restorative opportunities provided by UGS for urban residents [12,13].

Urbanization exerts a complicated influence on UGS [14,15,16,17]. Unplanned urban expansion decreases UGS area and degrades the quality of natural vegetation. The expansion of built-up areas generally destroys a great amount of the original vegetation cover and later replaces it with new artificial green patches, resulting in a high turnover rate for vegetation cover [18]. Vegetation in less developed regions is often more isolated and fragmented because economic growth and urbanization is regarded as a higher priority than maintaining urban vegetation [19]. However, in arid or semiarid cities, urbanization may result in an increase of vegetation by replacing primeval landscape with vegetated human settlements [20]. Since the mid-1980s, China has experienced arguably the most widespread and intensive urbanization in history, especially in the Yangtze River Delta, characterized not only by its enormous scale but also by unparalleled land use change [21]. More importantly, this process will not be halted in the short term. The latest National New-type Urbanization Plan of China projected that the proportion of permanent urban residents of China’s total population will rise from 35.7% in 2013 to reach 60% by 2020 [22]. The remaining green space within cities is increasingly recognized as being vital to the amelioration of urban living conditions [14,16].

Having realized the importance of urban green space, a number of global cities have set long-term strategies and policies to incorporate urban green space to help achieve a high-quality built environment. London has constructed a zonal greenbelt to shape patterns of urban development and create a green network by connecting green patches through a green wedge, green galleries, and channels to provide valuable habitat for flora and fauna [23]. Singapore has put forward the construction concept of being the “Garden City”, and set goals constantly in different development periods [24]. The Japanese government has practiced a controlled green land system which limits development area with laws and regulations. In addition, they have also adopted auxiliary systems such as loss compensation, land expropriation, and tax cuts to boost the enthusiasm of citizens to protect the green space [25]. Similarly, various green space planning strategies have been put in place by local governments to promote sustainable development [26,27].

There is great debate regarding the reliability and use of data approaches to quantify and track the changes, trends, and patterns of UGS over long periods. Owning to the increasing availability of image data from multiple sources, the quantification of spatiotemporal patterns for green space frequently relies on remote sensing [28,29,30]. However, data such as Lidar and high-resolution images are still not easily accessible for many regions or users due to the high costs of data acquisition. Moreover, it is usually impractical to provide full coverage of extensive metropolitan areas, with limited data available over long periods. With the advantages of global availability, repetitive data acquisition, and long-term consistency, Landsat series satellites have become the best compromise to overcome these limitations [14,18,26,31].

Green spaces within cities are generally highly fragmented and heterogeneous, characterized by abundant dispersed patches [31,32]. The commonly-used 30 m resolution Landsat data may fail to capture most of these small UGS patches (for example, lawns, street trees, residential yards), as well as their changes [30,33]. In recent years, multiple endmember spectral mixture analysis (MESMA) has provided a great advantage to overcoming this problem associated with using Landsat data to map urban components in highly heterogeneous urban environments [34,35,36,37].

Understanding the patterns of UGS is a complex issue, and utilizing landscape metrics analysis can be extremely helpful because of its ability to reveal the spatial features and changing processes in urban structures [38,39]. The size, accessibility, and connectivity of UGS are deemed to have a significant impact on the well-being of urban residents and the maintenance of biodiversity. Coupling remote sensing and landscape metrics analysis can provide valuable information on the spatial characteristics of UGS distribution and variation under urban development.

This study presents a new approach that combines sub-pixel mapping technology and landscape analysis with long-term time series Landsat images to describe the detailed pattern of UGS variation in a heterogeneous urban environment. The method involves two steps. First, MESMA was applied to time series Landsat images to estimate vegetation cover fraction (VCFs) at the sub-pixel level. Next, landscape metric analysis was applied to characterize the changing spatial-temporal pattern of the UGS. We implemented this approach to study Hangzhou during 1990, 2002, and 2013 in order to quantify the long-term spatiotemporal dynamics of UGS and explore its response to rapid urbanization and urban greening policy.

## 2. Methods

### 2.1. Study Site

Hangzhou lies about 180 km southwest of Shanghai and is one of the key cities within the Yangtze River Delta region. The study covers an area of around 3068 km^2^ with a population of 4.09 million. Hangzhou is a notable international garden city and one of China’s most popular tourist venues owing to its enchanting natural beauty and rich cultural heritage. With the rise of the Yangtze River Delta region as an emerging global urban region since the 1990s, Hangzhou has witnessed a rapid growth in economy and population [40]. This makes Hangzhou a typical case study for exploring the evolution of UGS associated with rapid urbanization.

### 2.2. Data and Preprocessing

Landsat series images have been widely used in urban studies [18,41]. The Landsat-8 OLI image obtained on 19 July 2013 and Landsat-5 TM images obtained on 23 September 2002 and 8 October 1990 were used to carry out the study. Orthorectified data with 30 m spatial resolution (Path: 119, Row: 39) were acquired from USGS [42], with special consideration of vegetation phenology. Subsequently, fast line-of-sight atmospheric analysis of spectral hypercubes (FLAASH) was applied to reduce atmospheric distortion and convert the digital numbers to surface reflectance. Finally, the study area was extracted based on the administrative boundary of the city. For endmember selection and accuracy assessment, two 0.5 m-resolution color aerial photographs acquired in 26 September 2002 and 17 August 2013 were collected from Google Earth.

### 2.3. Vegetation Fraction Retrieval

The MESMA procedure was implemented to correctly map the large number of small, fragmented vegetation patches that would otherwise be considered non-vegetated classes in medium resolution images [18]. The MESMA approach assumes that a landscape is formed from continuously varying proportions of several idealized surface materials called endmembers [34,43]. Its ability to incorporate a larger number of endmembers and allow the number and type of endmembers to vary for each pixel is critical for modeling urban components, especially in Chinese cities where a great variation of spectra materials exists due to rapid expansion and ubiquitous urban reconstruction [34,43,44,45].

All spectra collected from images were categorized into five endmembers: vegetation, impervious surfaces, soil, water, and shade. After that, a multi-complexity model procedure which contained two-, three-, and four-endmember models was applied to effectively and efficiently model the vegetation abundance in each pixel. Finally, the best-fit model for each pixel was chosen from a large number of candidate models via several criteria, and the vegetation fraction was calculated to generate vegetation coverage fraction maps. Accuracy assessment was conducted by comparing the VCFs with “true” vegetation coverage, visually interpreted from high-resolution aerial photographs. Two hundred random samples were generated for 2002 and 2013; each comprises an interpretation area of 90 × 90 m^2^. We only implemented accuracy assessments for 2002 and 2013 because a reference image for 1990 was unavailable. The coefficient of determination (R^2^), the root mean squared error (RMSE), and the systematic error (SE) were used to evaluate the accuracy and bias of derived VCFs [46]. Further details of this method can be found in our previous work [47].

### 2.4. Green Space Landscape Metric Analysis

Sub-pixel-based VCFs derived from MESMA possess physical meaning and provide a feasible way to examine the dynamics of the UGS pattern [48,49]. All the VCF images were uniformly reclassified into five-class thematic green space patches according to threshold: non-coverage (0–0.1), low coverage (0.1–0.3), medium coverage (0.3–0.6), high coverage (0.6–0.9), and full-coverage (0.9–1). Full-coverage pixels mainly represented green space patches such as large parks, vast lawns, farmland, and forests. The area of UGS patches was much larger than the pixel size (900 m^2^); therefore, each pixel was entirely covered by vegetation. Pixels designated as no coverage represented areas with little vegetation—usually impervious areas. Those labelled low coverage stood for areas containing small patches of vegetation, such as street and residential greening.

Based on the five-class green space coverage maps, a set of landscape metrics was calculated in FRAGSTATS 3.3 [50] to characterize the dynamic of UGS in consideration of domination, scattering, aggregation, and diversity. Five commonly-used landscape metrics (Table 1), including percentage of landscape (PLAND), patch density (PD), largest patch index (LPI), aggregation index (AI), and Shannon’s diversity index (SHDI), were applied to all coverage types as a whole (i.e., at the landscape level). Moreover, PLAND, PD, and LPI were also used to examine the spatial patterns of different coverage classes individually (i.e., at the class level).

**Figure 1:**
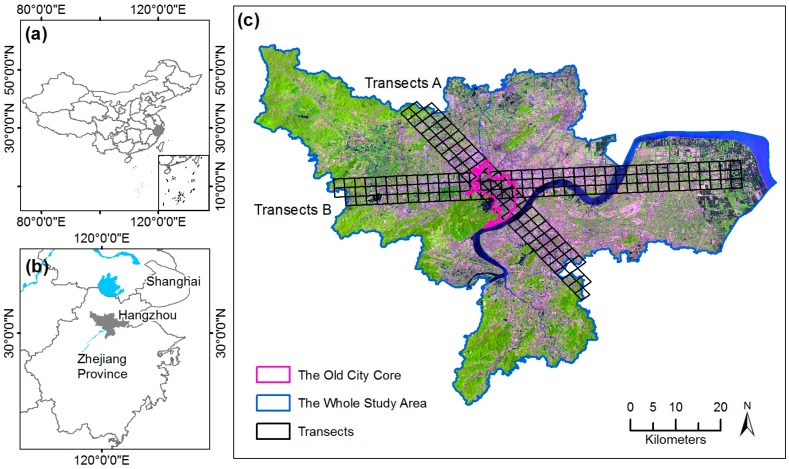
Maps showing (**a**) the location of Zhejiang Province in China; (**b**) the location of Hangzhou City; and (**c**) the study area, Hangzhou city, with the old city core and two study transects labeled.

The analysis was applied to the whole study area, including the old city core and urban–rural gradient. The boundary of the old city core was digitized through visual interpretation of the TM image of 1990. Additionally, two transects from north to south and west to east were used to derive the most significant changing pattern of UGS along the urban–rural gradient (Figure 1). Each transect had three adjacent rows, each 2 km wide. Both class- and landscape-level metrics were computed using a 3 × 3 (that is, 6 × 6 km^2^) overlapping moving window across the transect, in order to smooth out the noise caused by fine-scale and local variations [51].

## 3. Results

### 3.1. Accuracy Assessment

**Figure 2:**
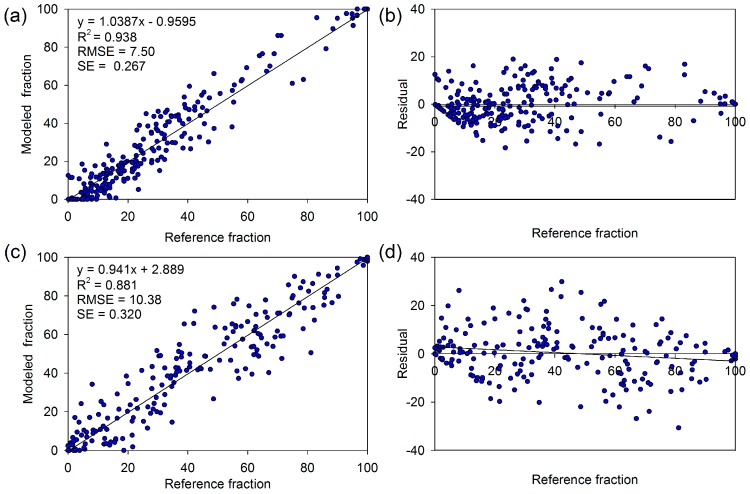
Results of the vegetation cover fraction (VCF) accuracy assessment for 2002 and 2013: (**a**) vegetation fraction scatter plot for 2013; (**b**) vegetation fraction residuals for 2013; (**c**) vegetation fraction scatter plot for 2002; and (**d**) vegetation fraction residuals for 2002.

The presented MESMA approach produced satisfactory accuracy in VCF. The RMSE was 7.50% and 10.38% for 2013 and 2002, respectively (Figure 2). Furthermore, the high R^2^ values ranged from 0.938 to 0.881, indicating a good fit between the modeled fractions and the “true” fractions. The low SE value (0.267% for 2013 and 0.320% for 2002) showed no significant under- or overestimation among the modeled VCFs.

**Figure 3:**
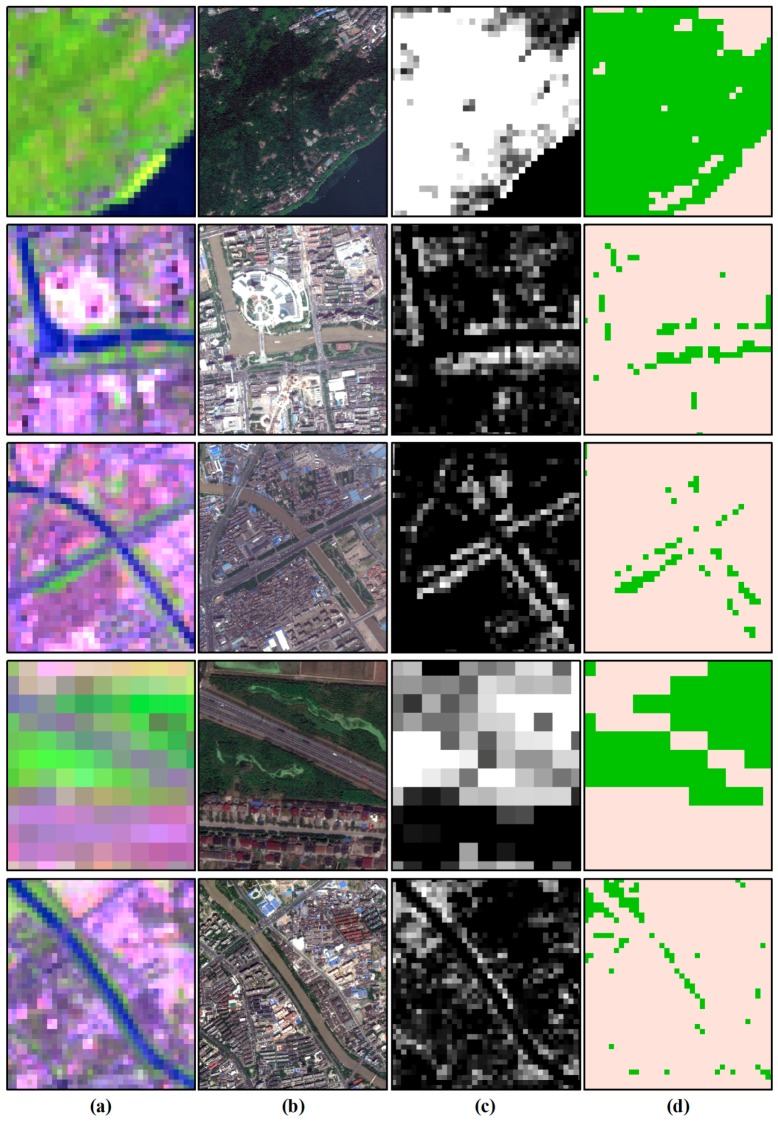
Comparisons between results from multiple endmember spectral mixture analysis (MESMA) and traditional pixel-based classification in 2013: (**a**) Landsat 8 OLI (RGB: 654; July 19, 2013); (**b**) high-resolution color-composition aerial photographs (August 17, 2013); (**c**) vegetation fraction from MESMA; and (**d**) vegetation classification result from SVM.

To further compare the MESMA procedure with the traditional pixel-based classification method, we applied support vector machine (SVM) classification to Landsat 8 OLI images from 2013 based on the spectra samples collected for MESMA (i.e., vegetation, soil, impervious, water), and merged the results into vegetation and non-vegetation categories (Figure 3). The MESMA approach and traditional pixel-based classifications such as SVM both work well for UGS patches of large areas (e.g., urban forest, large lawns, and farmland). However, pixels where vegetation covers were less than 100%—such as large green space patches with remaining bare soil—were overvalued by the traditional pixel-based classification method and simply categorized into the vegetation class. More importantly, only limited large UGS patches can be recognized by the traditional pixel-based classification method; the widespread small UGS patches such as residential yards, pocket parks, roadside trees, and green roofs were virtually ‘‘invisible’’ at worst, and greatly underestimated at best [33].

Because of the limited good-quality high-resolution images for 1990, we only implemented accuracy assessments for 2002 and 2013. The main factor affecting the accuracy of MESMA is the careful selection of representative spectra for endmembers. These spectra were generally selected from pixels representing pure land cover, such as from large patch urban forests, golf lawns, farms, and so on, which were relative easy to identify in Landsat images. Other parameters and procedures used in MESMA were consistent through the 3 years studied. Therefore, we assumed that the results from the 1990 fraction image would have acceptable accuracy if the vegetation fraction images for 2002 and 2013 were reasonably accurate. Nevertheless, the lack of an accuracy assessment for the 1990 fraction image is a limitation of this study.

### 3.2. General Dynamics of Green Space Landscape Pattern in Hangzhou City

**Figure 4:**
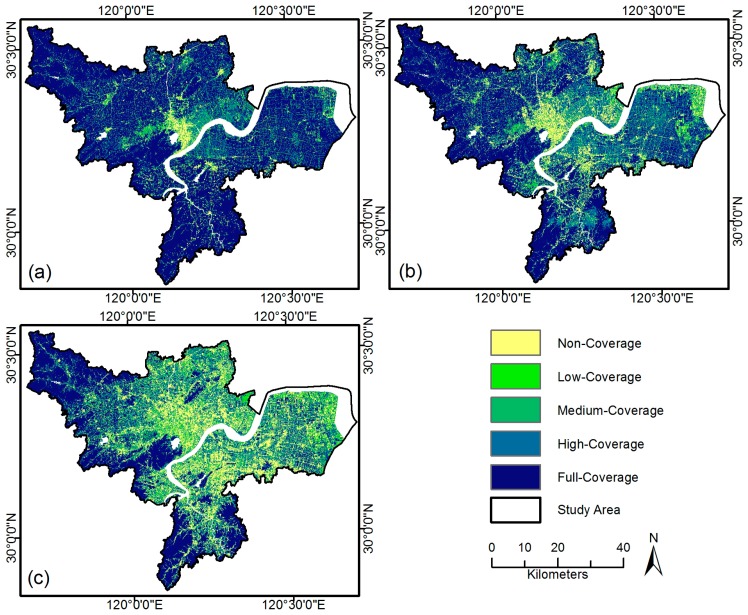
Vegetation coverage class maps of Hangzhou for (**a**) 1990, (**b**) 2002, and (**c**) 2013.

The green space landscape in Hangzhou experienced a drastic change in terms of composition and configuration in the past 20 years (Figure 4a–c). In 1990, full-coverage green space patches dominated and covered 69.76% of the study area. Since then, a continual reduction in UGS shrank sharply to 33.81% in 2013 (Table 2). Correspondingly, the PLAND for the non-coverage class rose from 4.5% in 1990 to 22.08% in 2013. Most green space losses occurred in peri-urban regions, mainly due to the expansion of built-up areas [40]. The most conspicuous change of LPI occurred in the full-coverage class, which declined sharply from 11.69 in 1990 to 4.05 in 2013, indicating heavy disturbances to full-coverage green space patches. Along with the urbanizing process, the predominant agriculture landscape was progressively replaced by an urban landscape, resulting in mixed land use and a chaotic landscape, as indicated by the increasing SHDI (Table 3). Meanwhile, the constant increase of PD and constant decrease of AI demonstrated a more fragmented and isolated vegetation landscape at the regional scale.

**Figure 5:**
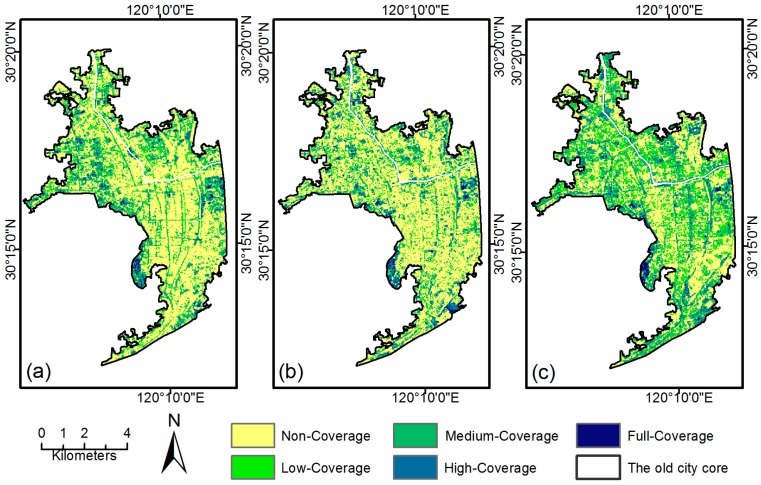
Vegetation coverage class maps of the city core for (**a**) 1990, (**b**) 2002, and (**c**) 2013.

Compared with the whole study area, the city core has a much higher proportion of non-coverage green space (Figure 5a–c). In 1990, about 56.42% of the old city center was covered by the non-coverage class, and there were very limited large green patches, most of which lay at the urban fringe and West Lake Scenic Spots. In contrast to the study area as a whole, there were no significant changes in UGS configuration observed in the first period; slight variations of PLAND, SHDI, and AI—indicating the greening policy in this period—showed very limited effect on the urban environment. Dominant non-vegetation coverage patches in the city core were enlarged due to infill development, as indicated by the peaking value of PLAND and LPI for non-coverage class in 2002.

A remarkable increase of PLAND for low- and medium-coverage classes was observed from 2002 to 2013, mainly due to emerging small-sized green patches in the city center. Meanwhile, the significant decrease of PLAND and LPI for the non-coverage class indicated that newly-built green space fragmented built-up areas that had little vegetation coverage before. The formation of green space networks resulted in a more diverse and heterogeneous UGS landscape, as evidenced by the significant decrease of AI and increase of SHDI.

### 3.3. Gradient Analysis of the Study Area Using Landscape Metrics

**Figure 6:**
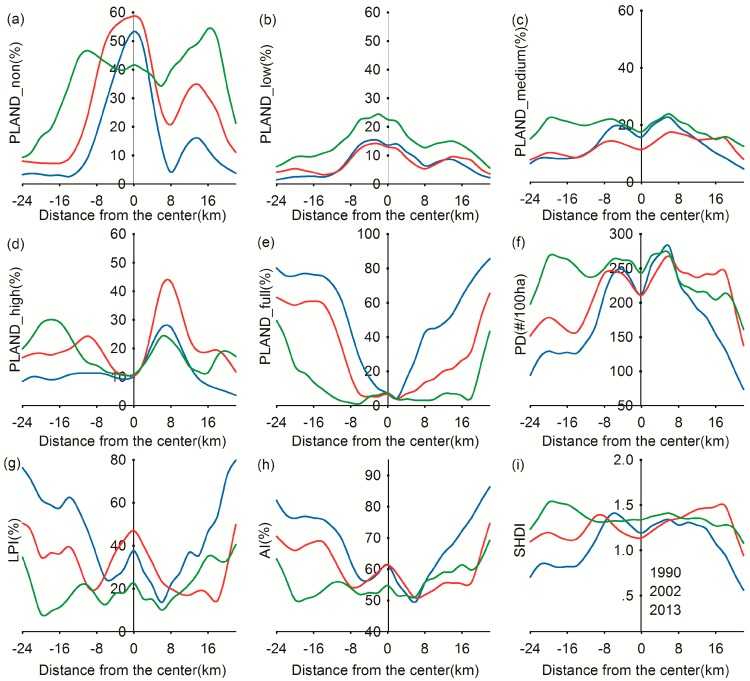
Gradient changes in selected metrics in the north to south transect in 1990, 2002, and 2013: (**a**) percentage of landscape for the non-coverage class; (**b**) percentage of landscape for the low-coverage class; (**c**) percentage of landscape for the medium-coverage class; (**d**) percentage of landscape for the high-coverage class; (**e**) percentage of landscape for the full-coverage class; (**f**) patch density (number of patches/100 ha); (**g**) largest patch index (%); (**h**) aggregation index (%); (**i**) Shannon’s diversity index. The values were obtained from each sampling cell from north to south.

Figure 6 shows the spatiotemporal change of hierarchical UGS patches along the transect over the past two decades. We presented only the result pertaining to the north–south transect in this section because the west–east and north–south transects showed similar patterns.

The changing of the relative dominance of hierarchical UGS patches indicated a changing land use pattern along the transect, shifting from natural landscape to artificial landscape. In 1990, the PLAND for the non-coverage class dominated in the urban center (−4 km to 4 km), accounting for nearly 50%, and there were very limited high-coverage and full-coverage classes. The PLAND for the full-coverage class gradually increased with distance, and became dominant on both ends of the transect. The increasing dominance of PLAND for the non-coverage class and the corresponding decrease of PLAND for the full-coverage class from 1990 to 2002 exhibit the hotspot of urban expansion in the northward direction. The Qiantang River lying to the south of the city center limited urban expansion, which explains the relatively insignificant change of vegetation coverage in this direction. During this period, most change was found in the urban periphery and was characterized by vegetation loss.

With the implementation of new urbanization strategies crossing the Qiantang River and developing southward, a very modern and rapidly developing sub city was built along the south bank of the Qiantang River. This is indicated by significantly increasing PLAND for the non-coverage class and decreasing PLAND for the full-coverage class, from 8 km to 16 km after 2002. Such a dramatic shift in vegetation coverage demonstrates the new hotspot of urban expansion in the southward direction. At the same time, a remarkable increase of PLAND for the low-coverage class appeared (from −8 km to 8 km), accompanied by the decreasing value of PLAND for the non-coverage class, indicating a conversion from non-coverage land to low-coverage land due to an increase of small-sized green patches in the city center.

In 1990, the LPI metric peaked at the urban center, fell swiftly in the urban fringe (−6 km and 6 km), and rose again in the outer region, with the increasing distance being a result of the full-coverage class gradually becoming dominant. Because of urban expansion, the remaining green space in the urban fringe was occupied, and more and larger patches of built-up area were formed, as indicated by the increased LPI in the urban area in 2002. However, with the new urban greening strategy in the latter decade, greatly increasing low- and medium-coverage green space fragmented the large built-up patches. Consequently, the vegetation landscape became more diverse, as evidenced by the significant decrease of LPI in most of the region in the 2013 transect.

The SHDI and AI calculated for the transect revealed spatial variation in different segments. In the city core, the increase of SHDI and decrease of AI from 2002 to 2013 indicated diversification and fragmentation of the vegetation landscape in the urban center. Taking into account the significant increase of PLAND for low- and medium-coverage classes, this reveals the recovery of UGS characterized by small-size green space patches. In the urban fringe, an initial decline was followed by a rise of SHDI, showing a fluctuating diversity because of infill development and the drastic loss of green space in the first period, with a recovery of UGS later. As for the outer region (8 km to 16 km), an opposing trend occurred due to urban sprawl in the first decade and the following infill development. This distinct pattern among different urban regions reflects the conversion process of vegetative landscape during urbanization, from natural vegetation to semi-natural and subsequently artificial green space.

## 4. Discussion

Despite its proven benefits, a widespread decline in UGS was observed in cities in the UK [16] and across Europe [52], particularly in most Eastern European cities [53]. Similarly, 17 of 20 major cities in the USA experienced a statistically significant decline in tree cover between 2003 and 2009 [54]. Recently, a study assessing UGS variation in 25 cities across the pan-Pacific region from 1984 to 2012 found that cities in China have the greatest vegetation loss, while cities in developed countries such as Canada, Japan, and the USA showed stable vegetation trends over time [20]. Based on statistical data released by the Chinese government, Zhao et al. [17] have indicated a steady increase in average UGC across 286 Chinese cities between 1989 and 2009. However, a study based on remotely-sensed normalized difference vegetation index (NDVI) data from 117 cities reported decreasing trends in UGS between 1982 and 2006 [55]. This conflicting result is mainly due to the difference in the definition and statistical caliber of UGS in two studies. The overall trend in the entire region shows lost vegetation cover; however, the old cities’ districts became greener in 2010 than they had been in 1990, and expanded built-up areas generally have higher UGC than old city districts [18].

Two distinct patterns identified in two periods in our study were largely due to the spatial characteristics of UGS patches and their changes. With the open policy of 1978, the Western ideal of the modern park was introduced to China. Based on the ideal of the western lake landscape, moderate and gradual point-by-point development was adopted to build the modern city park system [56]. Yet at the same time, under the significant pressure of a booming urban population and for the pursuit of economic benefits, a large amount of urban green space including even some newly-built parks was replaced by residential areas or commercial establishments [57]. These two opposing processes offset each other and led to an insignificant increase of UGS in 2002. The conflicts between booming demand for land, driven by urban population growth and land scarcity, severely restricted the amelioration of UGS. Except for a few newly-built urban parks, urban greening policy in this period showed very limited effect on the urban environment. On the contrary, dominant non-vegetation land patches in the old city center were enlarged by new emerging non-vegetation patches due to infill development.

The re-adjustment of the administrative boundary of Hangzhou in 2001 provided the city with more space to relieve its increasing population and building density, and additionally provides an opportunity for UGS construction [58]. Consequently, a green network system was designed, and a series of greening projects have been carried out since 2002. Meanwhile, new urban greening policies such as “stick in a pin wherever there’s room” and “borrow land to afforest” were gradually adopted to take full advantage of previously-neglected spaces to restore urban vegetation, especially in the city core [59]. These efforts included retrofitting green space in industrial brownfields and widespread tree planting alongside city streets, main roads, railway lines, and canals. In addition, the new Regulation of Hangzhou Urban Greening Management prescribed the level of greening coverage required in new built-up areas at no lower than 30% [60]. These new greening development strategies, with small-sized patches in the form of scattered distribution, contributed to the astonishing increase of low- and medium-coverage UGS in this period.

Providing sufficient public UGS has always been emphasized as a planning goal by the Chinese government. However, only the totality of UGS within a city was given emphasis during the policy-making process, neglecting the importance of its form and location [61]. In this study, we did not quantitatively analyze the variation in ecological quality, as it is almost unrealistic to accurately extract quality information on UGS (e.g., tridimensional greening quantity, vegetation composition, and species diversity) in Landsat data due to the mixed pixel problem. However, by analyzing the vegetation fraction variation within pixels, along with the derived landscape metrics, some features of UGS which are highly related to its ecological and social functions can be discussed. Most UGS loss in peri-urban regions was large patches of natural vegetation, causing the degradation of regional ecological quality. These larger UGS patches (e.g., urban forest) provided necessary habitats for biodiversity preservation and played important functions like carbon sequestration and energy conservation, which substantially influence urban dwellers from a regional and global perspective [9,62]. Meanwhile, the increase of UGS within the city was mostly in the form of scattered, small-size, artificial or semi-natural green space. These green spaces are unlikely to offer ecological services equivalent to the large natural vegetation patches, although they can also provide essential services that are critical to urban ecological functioning such as supporting the city birds’ diversity [63]. Additionally, large UGS patches are relatively rare and generally not within walking distance for most urban dwellers; in contrast, the abundant scatted small patches of UGS are widespread in places where people live, work, and play, and therefore provide a more evenly-distributed access to urban residents [64]. As cities grow, interactions between people and nature depend increasingly on green spaces like street plantings, backyards, and gardens for crucial cultural ecosystem services, such as providing important outdoor recreation opportunities for a variety of people [59], which are closely related to the quality of inner-city life [62,65].

In China, a spectacular real estate boom has pushed up the price of land [66]. Given the limited land available for greening in urban areas, utilizing neglected spaces—which are usually small-sized and fragmented—to improve urban greening has been widely adopted by local governments as an economical and effective way to increase green space [67]. It is challenging to measure these subtle and fragmented changes of UGS on medium-resolution data using traditional pixel-based classifiers. Despite its ability to detect the overall pattern, pixel-based classification fails to describe the abundance and subtle change of green space within a pixel, and therefore results in an unrealistic and inaccurate measurement of UGS [14]. However, information about the patterns of this kind of change might be crucial to complete and refine our understanding of the urban environmental evolution [33]. The VCFs derived from MESMA provide abundant information about vegetation coverage—especially isolated small-sized green space. Compared with interpretation based on high-resolution images, the disadvantage of the MESMA procedure is that this method could not give the actual location of the components within a pixel. By reclassifying the VCFs into different sub-pixel proportion classes, we were able to further apply landscape metrics to analyze the variation of UGS landscape. Although the achieved metric values are harder to interpret, we believe this is an alternative way to allow full play to the value of medium-resolution data in studying urban ecology.

With the advantages of freely-available worldwide repetitive data acquisition, spatial and spectral consistency over long-term periods, and stable quality, analyses of UGS variation based on our approach could be an important alternative approach to comparative studies of regional or even global urban environments.

## 5. Conclusions

Timely and accurate information about urban green space is crucial for urban environment management. However, the high spatial heterogeneity of urban environments challenges the mapping of UGS using medium-resolution data. The sub-pixel method proves to be a promising tool to overcome this problem and to provide a feasible and cost-effective way of exploring inter-urban green space dynamics. This paper provides comprehensive insight into the spatiotemporal dynamics of UGS landscapes during the rampant urbanization process in Hangzhou, China from 1990 to 2013. Despite complex spectral confusion in urban areas, the integration of MESMA and landscape analysis shows great ability to capture the spatial feature of UGS landscapes. Temporal changes of metrics facilitated the investigation and characterization of impacts on urban environments from accelerating urbanization.

Rapid urbanization over the past 20 years has brought about fundamental changes in green space composition and configuration. Green space landscapes also exhibit diverse spatiotemporal dynamics due to differences in urban growth and the implementation of green space protection policies. The whole study area has witnessed a significant loss of UGS, characterized by a significant increase of non-coverage patches. Most of these losses were located in peri-urban regions, mainly due to the expansion of built-up areas. In general, the regional UGS landscape has become more fragmented and isolated.

Yet unlike the study area as a whole, a remarkable amelioration of urban green space was observed in the inner-city core in the latter decade, in the form of the growth of low-coverage UGS patches as well as increasing patch diversity. Owing to new urban greening strategies in the second decade, a large amount of neglected space was utilized for urban greening. The green space network formed by newly-added small-sized vegetation has greatly fragmented the built-up areas and enriched the diversity of the urban environment. Our gradient analysis further revealed a distinct pattern of urban green space landscape variation in the process of urbanization, mainly a landscape conversion from natural vegetation to semi-natural and artificial green space. The spatiotemporal characteristics of the changing pattern of urban green space revealed the combined effects of rapid urbanization and greening policies.

This study demonstrates a practicable and meaningful application of time series Landsat images to explore the evolution of urban green space. Further integrated and cross-regional comparisons for cities with varying socioeconomic characteristics and natural conditions should be carried out to add to efforts for revealing, modeling, and predicting the change of urban environments in rapidly urbanizing cities. Our study highlighted the innovative efforts embraced by the local government to fully activate neglected spaces to restore green space within the urban area, given that there is generally very limited space for urban greening in rapidly growing high-density cities. Despite their small size, these green patches also provide essential ecological and social benefits by more evenly distributing access for most urban residents. Given increasing built-up areas and population density, broad strategies such as roof greening and vertical greening are suggested to keep up a high level of urban greening in the inner city. Green space patches should be preserved and enlarged during the process of urban redevelopment. Finally, conservation plans are still needed to protect forests and agricultural lands in suburban areas to serve as buffer zones to avoid potential loss caused by urbanization.

## Figures and Tables

**Table 1:** Descriptions of landscape metrics used in this study.

Landscape Metrics	Unit	Range	Justification
Percentage of landscape (PLAND)	Percent	0–100	A general index which depicts the relative abundance of each vegetation coverage type
Patch density (PD)	Number per 100 hectares	>0	Index of fragmentation
Largest patch index (LPI)	Percent	0–100	Index of fragmentation and dominance
Aggregation index (AI)	Percent	0–100	Index of spatial aggregation
Shannon’s diversity index (SHDI)	None	≥0	Index of diversity

**Table 2:** Spatial metrics for the whole study area and the old city core at class level.

Year	Class	The Whole Study Area			The Old City Core		
		PLAND	PD	LPI	PLAND	PD	LPI
1990	Non	4.50	8.00	0.43	56.42	19.42	22.88
	Low	3.80	23.46	0.00	17.00	91.97	0.09
	Medium	10.31	44.77	0.21	18.09	75.80	0.10
	High	11.63	48.32	0.03	7.05	39.06	0.14
	Full	69.76	7.28	11.69	1.44	5.80	0.08
2002	Non	11.40	17.81	0.76	59.84	20.98	29.87
	Low	4.91	26.63	0.01	14.58	87.31	0.04
	Medium	10.90	54.58	0.02	13.48	78.51	0.05
	High	22.72	46.88	0.22	10.81	38.61	0.25
	Full	50.06	14.80	11.80	1.30	5.82	0.06
2013	Non	22.08	19.76	0.69	41.77	31.46	5.60
	Low	9.54	46.10	0.03	24.95	84.21	0.20
	Medium	15.69	66.07	0.01	20.16	89.40	0.08
	High	18.87	45.14	0.05	11.43	48.36	0.08
	Full	33.81	13.42	4.05	1.69	7.25	0.31

**Table 3:** Spatial metrics for the whole study area and the old city core at landscape level.

Region	Year	Spatial Metrics			
		PD	LPI	AI	SHDI
The whole study area	1990	131.84	11.69	47.38	1.00
	2002	160.71	11.60	31.31	1.32
	2013	190.49	4.05	21.35	1.53
The city core	1990	232.06	22.88	57.27	1.18
	2002	231.23	29.87	56.82	1.16
	2013	260.68	5.60	50.67	1.35

## References

[1] Deng C.B., Wu C.S. (2013). Examining the impacts of urban biophysical compositions on surface urban heat island: A spectral unmixing and thermal mixing approach. Remote Sens. Environ..

[2] Liang Y., Jiang C., Ma L., Liu L., Chen W., Liu L. (2017). Government support, social capital and adaptation to urban flooding by residents in the Pearl River Delta area, China. Habitat Int..

[3] Xie Y.J., Ng C.N. (2013). Exploring spatio-temporal variations of habitat loss and its causal factors in the Shenzhen River cross-border watershed. Appl. Geogr..

[4] Bodnaruk E.W., Kroll C.N., Yang Y., Hirabayashi S., Nowak D.J., Endreny T.A. (2017). Where to plant urban trees? A spatially explicit methodology to explore ecosystem service tradeoffs. Landsc. Urban Plan..

[5] Davies R.G., Barbosa O., Fuller R.A., Tratalos J., Burke N., Lewis D., Warren P.H., Gaston K.J. (2008). City-wide relationships between green spaces, urban land use and topography. Urban Ecosyst..

[6] Tyrväinen L., Pauleit S., Seeland K., de Vries S., Konijnendijk C.C., Nilsson K., Randrup T., Schipperijn J. (2005). Benefits and uses of urban forest and trees. Urban Forests and Trees: A Reference Book.

[7] Akbari H., Pomerantz M., Taha H. (2001). Cool surfaces and shade trees to reduce energy use and improve air quality in urban areas. Sol. Energy.

[8] Pathak V., Tripathi B.D., Mishra V.K. (2011). Evaluation of Anticipated Performance Index of some tree species for green belt development to mitigate traffic generated noise. Urban For. Urban Green..

[9] Nowak D.J., Dwyer J.F. (2000). Understanding the Benefits and Costs of Urban Forest Ecosystems. Handbook of Urban and Community Forestry in the Northeast.

[10] Thompson C.W., Roe J., Aspinall P., Mitchell R., Clow A., Miller D. (2012). More green space is linked to less stress in deprived communities: Evidence from salivary cortisol patterns. Landsc. Urban Plan..

[11] Peters K., Elands B., Buijs A., Bell S. (2010). Social interactions in urban parks: Stimulating social cohesion?. Urban For. Urban Green..

[12] Donovan G.H., Butry D.T., Michael Y.L., Prestemon J.P., Liebhold A.M., Gatziolis D., Mao M.Y. (2013). The Relationship between Trees and Human Health: Evidence from the Spread of the Emerald Ash Borer. Am. J. Prev. Med..

[13] Everson-Rose S.A., Lewis T.T. (2004). Psychosocial factors and cardiovascular diseases. Annu. Rev. Public Health.

[14] Tang J.M., Chen F., Schwartz S.S. (2012). Assessing spatiotemporal variations of greenness in the Baltimore-Washington corridor area. Landsc. Urban Plan..

[15] Fuller R.A., Gaston K.J. (2009). The scaling of green space coverage in European cities. Biol. Lett..

[16] Dallimer M., Tang Z.Y., Bibby P.R., Brindley P., Gaston K.J., Davies Z.G. (2011). Temporal changes in greenspace in a highly urbanized region. Biol. Lett..

[17] Zhao J.J., Chen S.B., Jiang B., Ren Y., Wang H., Vause J., Yu H.D. (2013). Temporal trend of green space coverage in China and its relationship with urbanization over the last two decades. Sci. Total Environ..

[18] Yang J., Huang C., Zhang Z., Wang L. (2014). The temporal trend of urban green coverage in major Chinese cities between 1990 and 2010. Urban For. Urban Green..

[19] Grimm N.B., Faeth S.H., Golubiewski N.E., Redman C.L., Wu J., Bai X., Briggs J.M. (2008). Global change and the ecology of cities. Science.

[20] Lu Y., Coops N.C., Hermosilla T. (2017). Estimating urban vegetation fraction across 25 cities in pan-Pacific using Landsat time series data. ISPRS J. Photogramm Remote Sens..

[21] Deng J.S., Qiu L.F., Wang K., Yang H., Shi Y.Y. (2011). An integrated analysis of urbanization-triggered cropland loss trajectory and implications for sustainable land management. Cities.

[22] Bai X., Shi P., Liu Y. (2014). Society: Realizing China’s urban dream. Nature.

[23] Morrison N. (2010). A Green Belt under Pressure: The Case of Cambridge, England. Plan. Pract. Res..

[24] Tan P.Y., Wang J., Sia A. (2013). Perspectives on five decades of the urban greening of Singapore. Cities.

[25] Liu C., Shi T., Kohel A., Mlchio U. (2008). Retrospection on Policy Development of Urban Green Space in Japan and Apocalypse of Actual Urban Green Space from the Zoning System. Urban Plan. Forum.

[26] Zhou X.L., Wang Y.C. (2011). Spatial-temporal dynamics of urban green space in response to rapid urbanization and greening policies. Landsc. Urban Plan..

[27] Jim C.Y., Chen S.S. (2003). Comprehensive greenspace planning based on landscape ecology principles in compact Nanjing city, China. Landsc. Urban Plan..

[28] Patino J.E., Duque J.C. (2013). A review of regional science applications of satellite remote sensing in urban settings. Comput. Environ. Urban Syst..

[29] Mora B., Wulder M., White J., Hobart G. (2013). Modeling Stand Height, Volume, and Biomass from Very High Spatial Resolution Satellite Imagery and Samples of Airborne LiDAR. Remote Sens..

[30] Zhou W., Troy A., Grove M. (2008). Object-based Land Cover Classification and Change Analysis in the Baltimore Metropolitan Area Using Multitemporal High Resolution Remote Sensing Data. Sensors.

[31] Li X.M., Zhou W.Q., Ouyang Z.Y. (2013). Relationship between land surface temperature and spatial pattern of greenspace: What are the effects of spatial resolution?. Landsc. Urban Plan..

[32] Qian Y., Zhou W., Li W., Han L. (2015). Understanding the dynamic of greenspace in the urbanized area of Beijing based on high resolution satellite images. Urban For. Urban Green..

[33] Qian Y., Zhou W., Yu W., Pickett S.T.A. (2015). Quantifying spatiotemporal pattern of urban greenspace: New insights from high resolution data. Landsc. Ecol..

[34] Liu T., Yang X.J. (2013). Mapping vegetation in an urban area with stratified classification and multiple endmember spectral mixture analysis. Remote Sens. Environ..

[35] Michishita R., Jiang Z.B., Xu B. (2012). Monitoring two decades of urbanization in the Poyang Lake area, China through spectral unmixing. Remote Sens. Environ..

[36] Powell R.L., Roberts D.A., Dennison P.E., Hess L.L. (2007). Sub-pixel mapping of urban land cover using multiple endmember spectral mixture analysis: Manaus, Brazil. Remote Sens. Environ..

[37] Feng Q., Gong J., Liu J., Li Y. (2015). Flood Mapping Based on Multiple Endmember Spectral Mixture Analysis and Random Forest Classifier—The Case of Yuyao, China. Remote Sens..

[38] Zhang Z., Su S., Xiao R., Jiang D., Wu J. (2013). Identifying determinants of urban growth from a multi-scale perspective: A case study of the urban agglomeration around Hangzhou Bay, China. Appl. Geogr..

[39] Su S., Wang Y., Luo F., Mai G., Pu J. (2014). Peri-urban vegetated landscape pattern changes in relation to socioeconomic development. Ecol. Indic..

[40] Deng J.S., Wang K., Hong Y., Qi J.G. (2009). Spatio-temporal dynamics and evolution of land use change and landscape pattern in response to rapid urbanization. Landsc. Urban Plan..

[41] Buyantuyev A., Wu J., Gries C. (2010). Multiscale analysis of the urbanization pattern of the Phoenix metropolitan landscape of USA: Time, space and thematic resolution. Landsc. Urban Plan..

[42] USGS Global Visualization Viewer. http://glovis.usgs.gov/.

[43] Roberts D.A., Gardner M., Church R., Ustin S., Scheer G., Green R.O. (1998). Mapping chaparral in the Santa Monica Mountains using multiple endmember spectral mixture models. Remote Sens. Environ..

[44] Lu D.S., Weng Q.H. (2004). Spectral mixture analysis of the urban landscape in Indianapolis with landsat ETM plus imagery. Photogram. Eng. Remote Sens..

[45] Lu D.S., Batistella M., Moran E. (2004). Multitemporal spectral mixture analysis for Amazonian land-cover change detection. Can. J. Remote Sens..

[46] Wu C.S., Yuan F. (2007). Seasonal sensitivity analysis of impervious surface estimation with satellite imagery. Photogram. Eng. Remote Sens..

[47] Gan M., Deng J., Zheng X., Hong Y., Wang K. (2014). Monitoring Urban Greenness Dynamics Using Multiple Endmember Spectral Mixture Analysis. PLoS ONE.

[48] Small C. (2001). Estimation of urban vegetation abundance by spectral mixture analysis. Int. J. Remote Sens..

[49] Elmore A.J., Mustard J.F., Manning S.J., Lobell D.B. (2000). Quantifying Vegetation Change in Semiarid Environments: Precision and Accuracy of Spectral Mixture Analysis and the Normalized Difference Vegetation Index. Remote Sens. Environ..

[50] McGarigal K., Cushman S., Neel M.C., Ene E. (2002). FRAGSTATS v3: Spatial Pattern Analysis Program for Categorical Maps. http://www.umass.edu/landeco/research/fragstats/fragstats.html.

[51] Luck M., Wu J. (2002). A gradient analysis of urban landscape pattern: A case study from the Phoenix metropolitan region, Arizona, USA. Landsc. Ecol..

[52] Baycan-Levent T., Vreeker R., Nijkamp P. (2009). A Multi-Criteria Evaluation of Green Spaces in European Cities. Eur. Urban Reg. Stud..

[53] Kabisch N., Haase D. (2013). Green spaces of European cities revisited for 1990–2006. Landsc. Urban Plan..

[54] Nowak D.J., Greenfield E.J. (2012). Tree and impervious cover change in US cities. Urban For. Urban Green..

[55] Sun J., Wang X., Chen A., Ma Y., Cui M., Piao S. (2011). NDVI indicated characteristics of vegetation cover change in China’s metropolises over the last three decades. Environ. Monit. Assess..

[56] Gao Y. (2012). The Influence of Western Landscape Architecture Art on Modern Hangzhou Parks. Master’s Thesis.

[57] Li G. (2006). A Study of the Development History of Westlake Landscape in Hangzhou. Master’s Thesis.

[58] Wu K., Zhang H. (2012). Land use dynamics, built-up land expansion patterns, and driving forces analysis of the fast-growing Hangzhou metropolitan area, eastern China (1978–2008). Appl. Geogr..

[59] Wolch J.R., Byrne J., Newell J.P. (2014). Urban green space, public health, and environmental justice: The challenge of making cities ‘just green enough’. Landsc. Urban Plan..

[60] Yuan Y., Han Y., Zhang Z., Liu Y. (2015). Research on zoning of green area ratio in residential area: Based on panyu district of Guangzhou City. City Plan. Rev..

[61] Kong F.H., Nakagoshi N. (2006). Spatial-temporal gradient analysis of urban green spaces in Jinan, China. Landsc. Urban Plan..

[62] Heynen N.C. (2003). The Scalar Production of Injustice within the Urban Forest. Antipode.

[63] Sandström U.G., Angelstam P., Mikusiński G. (2006). Ecological diversity of birds in relation to the structure of urban green space. Landsc. Urban Plan..

[64] Peschardt K.K., Schipperijn J., Stigsdotter U.K. (2012). Use of Small Public Urban Green Spaces (SPUGS). Urban For. Urban Green..

[65] Niemelä J. (2014). Ecology of urban green spaces: The way forward in answering major research questions. Landsc. Urban Plan..

[66] Dreger C., Zhang Y. (2013). Is there a Bubble in the Chinese Housing Market?. Urban Policy Res..

[67] Byrne J.A., Lo A.Y., Yang J. (2015). Residents’ understanding of the role of green infrastructure for climate change adaptation in Hangzhou, China. Landsc. Urban Plan..

